# A Microdroplet Fluorescence Method for the Determination of Nitrite in Water Using a Hydrophobic Cellulose Substrate Platform

**DOI:** 10.1021/acsomega.6c06452

**Published:** 2026-09-29

**Authors:** Júlia C. Romero, Vanesa Romero, Carlos Bendicho, Fábio A. C. Amorim

**Affiliations:** † Departamento de Ciências Exatas, Universidade Estadual de Santa Cruz, Campus Soane Nazaré de Andrade, Rodovia Jorge Amado, Ilhéus 45662-900, Brazil; ‡ Centro de Investigación Mariña, Departamento de Química Analítica y Alimentaria, Grupo QA2, Edificio CC Experimentais, 16784 Universidade de Vigo, Campus de Vigo, As Lagoas Marcosende, Vigo 36310, Spain

## Abstract

In this work, a miniaturized and sustainable method was developed, based on the fluorimetric reaction with 2,3-diaminonaphthalene (DAN) in an acid medium, for the determination of trace amounts of nitrite in different water samples using a hydrophobic cellulose substrate platform. The innovation consists of the use of a nonwettable cellulose substrate that suspends stable microdroplets, enabling direct measurement of fluorescence on the front surface and mitigating the signal attenuation inherent in traditional hydrophilic paper devices. The cellulose substrate acted both as a solid support for the reaction and as a holder for microdroplet suspension during fluorescence signal acquisition. The method was designed to reduce reagent consumption and waste generation by operating at the microliter scale, using a total reaction volume of 15 μL. The procedure showed great tolerance to the presence of 11 potentially interfering ions, evaluated at concentrations relevant to water samples. The values obtained for the limit of detection were 0.35 μg L^–1^, the limit of quantification was 1.17 μg L^–1^. The precision, based on the relative standard deviation (RSD%), was 3.5% (*N* = 7). The method was successfully applied to various natural waters, with recoveries ranging from 83 to 115%. With an AGREEprep score of 0.77, the procedure significantly reduces reagent consumption and waste, offering an environmentally friendly and low-cost alternative to conventional laboratory assays and providing a simple and sensitive alternative for laboratory-based environmental monitoring of nitrite.

## Graphical abstract

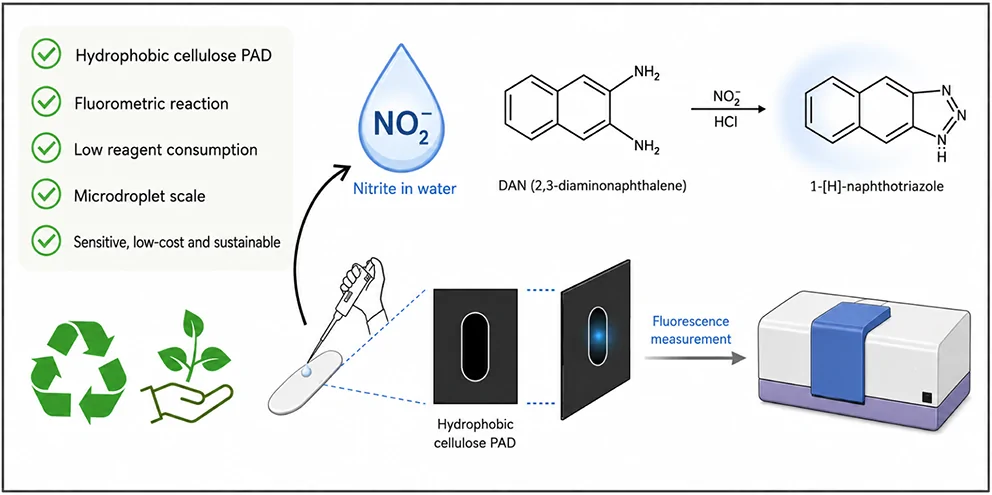

## 1 Introduction

Nitrite is one of the substances responsible for water pollution. It can occur naturally through the nitrogen cycle in ecosystems or because of anthropogenic activities, such as the use of nitrogen-based fertilizers. Other causes of nitrite contamination include human, animal, and industrial waste.^1^ Nitrite present in the human body can lead to adverse health effects, such as methemoglobinemia, a condition that impairs the blood’s ability to transport oxygen.^2^ In addition, nitrite can be converted into nitrosated compounds, such as *N*-nitrosamines, which are potential carcinogenic compounds in the digestive system.^3^ Due to the health risks associated with nitrite exposure, several international, national, and regional organizations have established regulatory limits for nitrite concentrations in drinking water. The World Health Organization (WHO) has established a provisional guideline value of 0.2 mg L^–1^ for long-term exposure to nitrite, while maintaining a 3 mg L^–1^ limit for short-term exposure.^4^ In contrast, the European Union (EU) has established a maximum allowable concentration of 0.5 mg L^–1^.^5^

Different analytical techniques have been reported in the literature for the determination of nitrite in water samples, including ion chromatography,^6^ capillary electrophoresis,^7^ electrochemistry,^8^ resonance Rayleigh scattering,^9^ spectrofluorimetry,^10^ chemiluminescence,^11^ spectrophotometry,^12^ and smartphone-based colorimetry.^13^ Among them, the most popular is the Griess method, which involves a color-forming diazotization reaction that allows detection by spectrophotometry or colorimetry. However, this method lacks high sensitivity with a detection limit in the micromolar range, which hinders its application in trace and ultratrace-level analysis. Since nitrite can be found at very low concentrations in environmental matrices, the development of more sensitive analytical methods is of high interest. In this context, fluorescence-based assays have gained increasing attention because of their superior sensitivity, selectivity, and potential for integration into portable equipment. In addition to this approach, fluorescence spectroscopy can also be employed on the basis of reactions with fluorescent compounds.^14,15^

In this context, the reaction between nitrite and 2,3-diaminonaphthalene (DAN) has been widely employed in fluorometric analysis for decades.^16^ This method is based on the diazotization of one of DAN’s amino groups by the nitrite ion under acidic conditions, resulting in the formation of the fluorescent product, i.e., 1-[H]-naphthotriazole.^17^ This compound is the main contributor to fluorescence emission, and it remains stable under basic conditions, enabling the quantification of nitrite at low concentrations.^18^ A paramount trend in modern analytical chemistry focuses on the development of environmentally friendly, low-cost, simple, and rapid methods that can be implemented at reduced scales.^19,20^ In this context, paper-based materials have gained considerable attention as versatile platforms for analytical applications. Cellulose-based substrates offer several advantages, including wide availability, biodegradability, biocompatibility, low cost, and ease of disposal. In addition, they are compatible with a broad range of chemical substances. These characteristics make them suitable supports for the development of miniaturized analytical systems aimed at the determination of chemical species of interest.^21^

Usually, the fabrication of paper-based materials relies on hydrophilic cellulose substrates, whose natural wicking properties enable liquid transport and a straightforward fabrication. Its application in chemical analysis allows reaction reagents and products to penetrate and become dispersed within the substrate, which can lead to a reduction in the analytical signal in colorimetric and fluorometric analyses.^20^ Moreover, hydrophobic cellulose (i.e., water-proof cellulose) has recently emerged as an attractive solid support for developing paper-based devices, since its nonwetting surface can hold stable microdroplets, enabling its application in microextraction and sensing. Furthermore, the use of hydrophobic cellulose substrates allows for low-cost miniaturization of analytical approaches. The easy handling and great robustness of these solid substrates open the possibility of performing direct measurements with conventional instrumentation, including localized measurements such as spectrophotometric or spectrofluorimetric detection, avoiding the use of expensive microvolume cuvettes. In addition, the low wettability of the hydrophobic substrates helps minimize attenuation of the analytical signal, whether colorimetric or luminescent, which is typically one of the main drawbacks of hydrophilic cellulose substrates, where reagents become embedded within the white cellulose matrix. By enabling the reaction to occur on the surface rather than within the bulk material, the resulting color intensity or luminescence emission becomes enhanced, thereby improving the overall sensitivity of the assay.^22^

In this work, a sustainable, miniaturized approach for determining nitrite in water samples by fluorescence sensing is proposed. The novel approach is based on confining the classic fluorimetric reaction between nitrite and 2,3-diaminonaphthalene (DAN) to an acidic medium within a microdroplet, supported on a hydrophobic cellulose substrate. Although the fluorometric reaction using DAN for nitrite determination is well established for conventional in-solution analyses, no studies have reported its combined application with hydrophobic cellulose substrates as analytical platforms. In the proposed procedure, the cellulose substrate acts not only as a support but also as a microdroplet suspension platform that allows for the confinement of reagents, reduced reagent consumption, and the direct acquisition of the fluorescence signal using conventional instrumentation without additional transfer steps. The layout of the cellulose substrate served both as the solid support for carrying out the sensing reaction and as the platform for suspending the microdroplet during direct fluorescence signal acquisition, contributing to the development of simpler and more sustainable analytical approaches.

## 2 Experimental Section

### 2.1 Instruments, Materials, and Reagents

Front-face fluorescence measurements were performed by means of a spectrofluorometer Horiba model Fluoromax-4 series (Tokyo, Japan) equipped with a solid sample holder model J1933 with an incidence angle of excitation of 60°. An excitation wavelength of 365 nm and an emission wavelength of 405 nm were used. A temperature-controlled environment (1 to 15 °C) was employed to maintain constant fluorescence reaction conditions. Whatman grade 1PS hydrophobic filter paper (Cytiva, Shanghai, China), made up of cellulose impregnated on its surface with a stabilized silicone, was used as the hydrophobic cellulose-based microdroplet support for sensing reaction and fluorescence analysis. A vacuum filtration system and 0.45 μm cellulose nitrate membrane filters were used for filtering water samples prior to analysis.

For the fluorometric reaction, 2,3-diaminonaphthalene (DAN) (TCI, Tokyo, Japan), 37% (m/m) hydrochloric acid (VWR Prolabo, Fontenay–sous–Bois, France), sodium hydroxide (VWR Prolabo, Fontenay–sous–Bois, France), and ethylenediaminetetraacetic acid (EDTA) (Sigma-Aldrich, Steinheim, Germany) were employed. Nitrite standard solutions were prepared daily from sodium nitrite (Sigma-Aldrich, Steinheim, Germany) previously dried at 120 °C for 1 h. Nitrite working solutions were made up by dilution with ultrapure water to concentrations from 10 to 150 μg L^–1^. DAN solution was prepared by dissolving the commercial reagent in 0.25 mol L^–1^ HCl solution. A 1000 mg L^–1^ DAN stock solution was prepared weekly and stored at 4 °C, protected from light. DAN working solutions were prepared daily by dilution with 0.25 mol L^–1^ HCl solution.

All reagents, including salts used for interference tests, were of an analytical grade. Ultrapure water was obtained from a Merck Millipore Simplicity water purification system (Darmstadt, Germany) and was used throughout the experiments.

### 2.2 Sampling

All samples were collected on the day of analysis using clean and dry polypropylene containers. The samples were kept refrigerated, following analytical quality standards for sample preservation. A tap water sample was collected at the University of Vigo. Samples from a public drinking fountain (42°12′41.8″ N, 8°43′53.54″ W), a natural spring water fountain (42°12′51.43″ N, 8°43′45.13″ W), a wall-mounted public drinking fountain (42°13′35.2″ N, 8°43′15.05″ W), river water (42°9′30.62″ N, 8°41′31.59″ W), and reservoir water (42°9′33.9″ N, 8°41′36.32″ W) were collected in the city of Vigo, Spain. Commercial mineral water samples were obtained from a local market.

All water samples were filtered using a vacuum filtration system with 0.45 μm cellulose nitrate membrane filters and maintained at 4 °C prior to analysis.

### 2.3 Assembly of the Hydrophobic Cellulose Substrate Platform and Front-Face Fluorescence Measurements

The platform, based on a hydrophobic cellulose substrate, employed as the reaction zone and sample support, was prepared by manually cutting Whatman 1PS filter paper using an elliptical template with a size of 6 mm in width and 13 mm in length (Figure 1A). The substrate is commercially available and was used as received without any additional chemical modification, ensuring that its original surface properties were maintained. This substrate size and shape fit precisely within the central area of the solid sample holder for performing the front-face fluorescence measurements (Figure 1B). The reaction mixture containing the sample, in microdroplet form, was deposited at the center of the paper substrate to ensure that the excitation beam effectively reached the sample, allowing proper alignment with the optical path of the spectrofluorimeter. After the reaction, the hydrophobic cellulose substrate platform was placed vertically into the sample holder.

**Figure 1:**
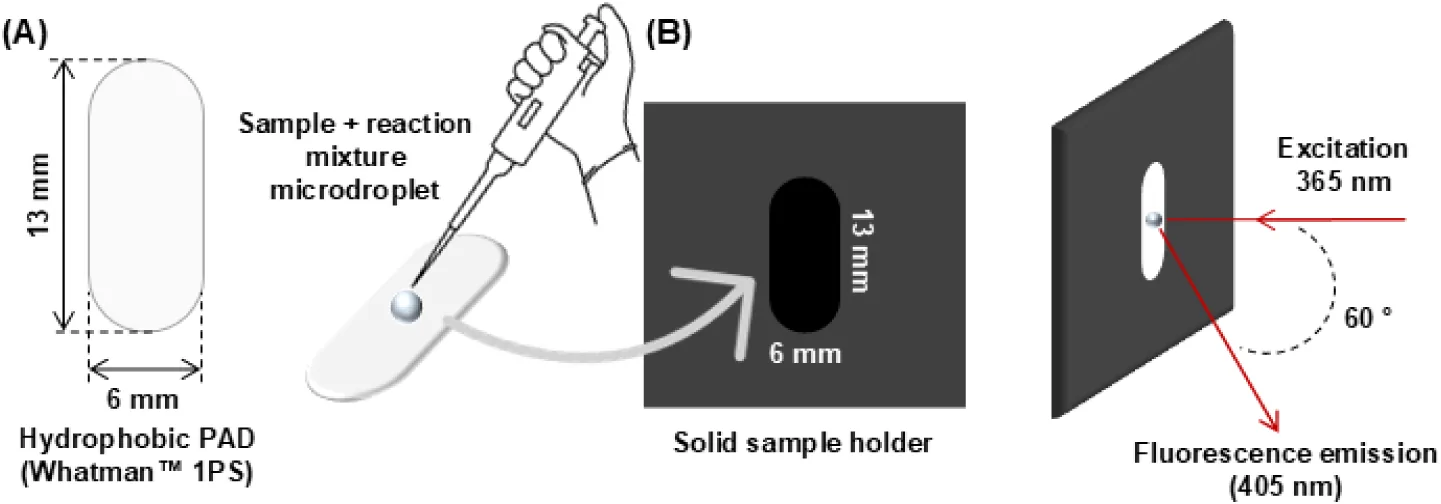
Hydrophobic cellulose substrate size and shape (A). Solid sample holder and fluorescence analysis display (B).

Preliminary stability tests indicated that microdroplets remain firmly adhered to the Whatman 1PS substrate even when the platform was completely inverted (180°). This high level of adhesion, characteristic of the silicone-impregnated cellulose surface, as previously reported,^22^ ensures the absolute stability and integrity of the droplets during the required vertical alignment. The microdroplet remains suspended and stable for fluorescence measurement, which was performed using the fixed geometry of the spectrofluorimeter, with a 60° angle between the excitation and emission beams. The physical arrangement of the platform and the visual stability of the suspended microdroplet in the solid-sample holder are shown in Figure S1 (Supporting Information).

### 2.4 Experimental Procedure for Nitrite Determination

For nitrite determination, a 10 μL aliquot of water sample, nitrite standard solution, or blank was placed on the center of the hydrophobic cellulose substrate. Then, 1 μL of 0.30 mol L^–1^ EDTA and 2 μL of 50 mg L^–1^ DAN were added. The hydrophobic cellulose substrate containing the reaction mixture was placed in a dark cabinet protected from light in a refrigerated environment at 10 °C. After 10 min, 2 μL of 0.50 mol L^–1^ sodium hydroxide was added to reach alkaline pH medium in the microdroplet, thus ensuring a stable and high fluorescence signal for nitrite determination. The reagents were mixed carefully by repeated aspiration and dispensing cycles using a micropipette to ensure proper homogenization of the microdroplet. Finally, the hydrophobic cellulose substrate was placed in the sample holder (incidence angle of excitation = 60°), and the analytical response, as fluorescence intensity, was registered. All measurements were done in triplicate.

### 2.5 Analytical Performance

To validate the procedure, the following analytical performance parameters were evaluated: limit of detection (LOD), limit of quantification (LOQ), precision (repeatability), and accuracy through spiking and recovery tests of the analytes in the samples. Interference tests were also performed for K^+^, Ca^2+^, Mg^2+^, Fe^2+^, Fe^3+^, Zn^2+^, F^–^, NO_3_^–^, HCO_3_^–^, SO_4_^2–^, and SO_3_^2–^.

The limit of detection (LOD) was determined based on three times the standard deviation of the analytical blank signal divided by the slope of the analytical curve, defined as LOD = 3s/b (where s is the standard deviation obtained from the signal of 10 blank measurements and b is the slope of the analytical curve), and the limit of quantification (LOQ) was determined as ten times the standard deviation of the blank signal divided by the slope of the analytical curve. These values were calculated in μg L^–1^.

## 3 Results and Discussion

### 3.1 Fluorescence Response Mechanism

The reaction between 2,3-diaminonaphthalene (DAN) and nitrite proceeds through acid-catalyzed nitrosation, where nitrite is protonated to form nitrous acid that generates the electrophilic nitrosonium ion (NO^+^) (Figure 2a–b). This ion selectively nitrosates the protonated amino group of DAN, enabling intramolecular cyclization to yield 1-[H]­naphthotriazole (Figure 2c). In an acidic medium, the triazole product is protonated, which quenches its fluorescence. Thus, before fluorescent measurement, the reaction product must be rendered in an alkaline medium (pH 8.5–12), ensuring triazole product deprotonation (Figure 2d). High pH restores full conjugation within the aromatic structure, exhibiting higher fluorescence intensity and greater stability, with a characteristic emission peak at 405 nm^23,24^ (see Figure S2, Supporting Information).

**Figure 2:**
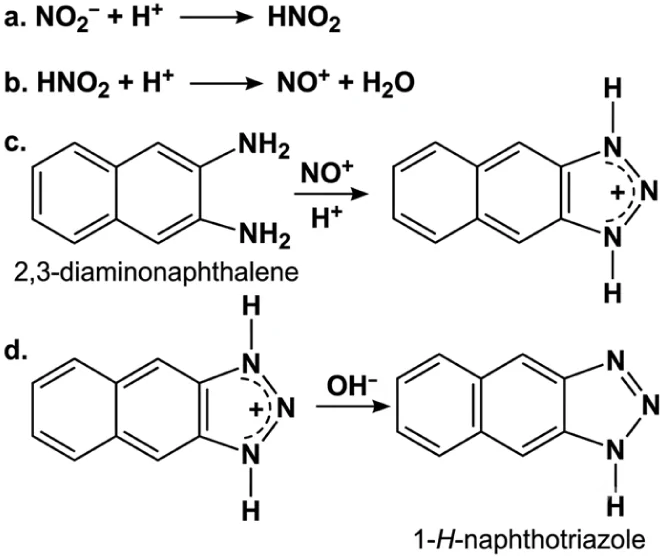
Reaction scheme for the fluorescence sensing of nitrite with DAN. (a–b) Acid-catalyzed formation of the electrophilic nitrosonium ion (*NO*+) from nitrite; (c) selective nitrosation of the amino group of DAN followed by intramolecular cyclization to form 1-[H]-naphthotriazole; and (d) deprotonation of the triazole product in alkaline medium (pH 8.5–12.0) to restore full aromatic conjugation and achieve maximum fluorescence intensity.

### 3.2 Preliminary Studies

The initial parameters were established through screening studies. As explained before, controlling the reaction pH is crucial for both the nitrosation step and subsequent fluorescence measurement of the triazole product. Studies indicate that the optimal reaction pH for nitrosation ranges from 1 to 2. Furthermore, the triazole product reaches maximum fluorescence intensity at a pH between 8.5 and 12.^25^ Thus, using 2 μL of DAN reagent in 0.25 mol L^–1^ HCl ensured the recommended pH for these reaction stages. The volume of 2 μL and the concentration of NaOH (0.50 mol L^–1^) were rigorously adjusted and experimentally verified using pH indicator paper to ensure the rapid transition from the acidic reaction medium to the alkaline detection medium (pH between 10 and 11), a condition for the maximum emission of 1-[H]-naphthotriazole.

Besides, the addition of 1 μL of 0.30 mol L^–1^ EDTA in the reaction medium improved method selectivity without affecting sensitivity. This concentration was established to ensure the complexation of potentially interfering metal ions frequently found in water samples. Higher EDTA concentrations were also evaluated but did not promote additional selectivity gains.

A sample or standard solution volume of 10 μL was considered satisfactory for microanalysis applications, defined as the upper operational limit, which, added to the reagents (totaling 15 μL). Given the reduced size and hydrophobic properties of the cellulose substrate, only limited reagent volumes could be used, as the microdroplet remains vertically positioned during analysis. A total volume of 15 μL corresponded to the upper limit of microdroplet stability, allowing the physical integrity of the system to be maintained during vertical positioning in the spectrofluorometer. Larger volumes resulted in microdroplet instability and a higher risk of liquid overflow onto the hydrophobic surface, while smaller volumes reduced the area of incidence of the excitation beam, compromising the intensity of the analytical signal. This volume proved effective and was adopted for optimization studies.

### 3.3 Optimization of Experimental Parameters

Different parameters involved in the fluorometric reaction were optimized, including reaction temperature, DAN reagent concentration, and reaction time. The decision to perform univariate optimization was based on the need to understand the individual behavior of each variable under the conditions of reaction miniaturization on the hydrophobic cellulose substrate. Although the reaction between nitrite and DAN is well established for conventional systems in solution, its adaptation to a microscale environment posed challenges related to microdroplet stability, evaporation, and surface distribution of the reagents, requiring a detailed assessment of the influence of each variable on the fluorescent signal. Optimization experiments were carried out by triplicate using 50 μg L^–1^ nitrite. The analytical response is expressed as fluorescence signal intensity (FI), measured in Counts Per Second (CPS).

#### 3.3.1 Effect of Reaction Temperature

The temperature of the reaction medium can affect the performance, kinetics, and efficiency of a chemical reaction.^26^ Additionally, temperature directly influences fluorescence intensity, independent of the reaction characteristics.^27^ Temperatures ranging from 4 to 40 °C were studied (Figure 3a). The fixed parameters were a reaction time of 10 min and a DAN concentration of 50 mg L^–1^.

**Figure 3:**
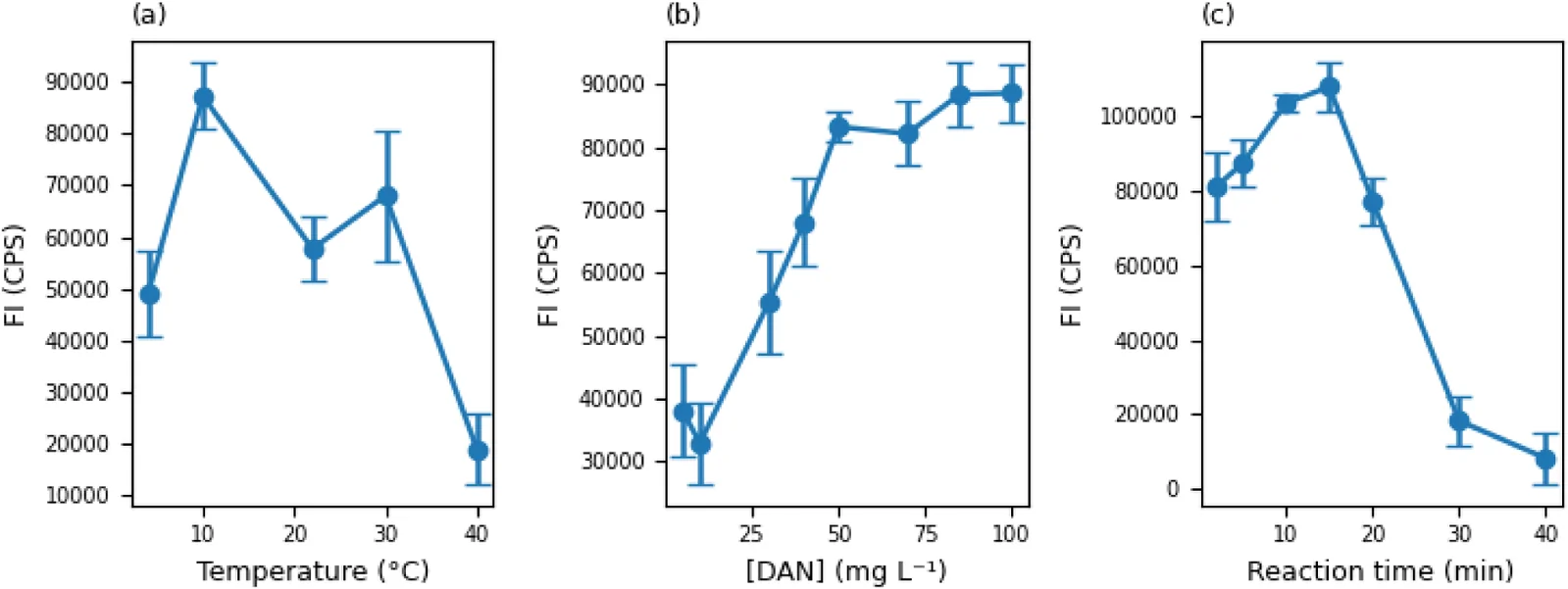
Optimization of reaction temperature (a); DAN concentration (b); reaction time (c).

In miniaturized microdroplet-based systems, temperature control becomes even more critical due to the greater influence of phenomena such as evaporation and fluorescence signal instability compared to conventional macroscale systems. As shown in Figure 3a, the analytical response increased considerably at 10 °C and showed minor variations at the other temperatures studied. Literature reports indicate that quantum yield, and consequently fluorescence intensity, increases at lower temperatures.^27^ Moreover, the lifetimes of fluorescent molecules in the excited state are longer at reduced temperatures. This type of low-temperature reaction ensures the stability of the fluorescent product and can prevent the degradation that occurs at higher temperatures.^28^ In accordance with these results, a reaction temperature of 10 °C is chosen for further experiments, ensuring a better analytical signal and greater microdroplet stability.

#### 3.3.2 Effect of 2,3-Diaminonaphthalene (DAN) Concentration

The concentration of DAN influences the reaction with nitrite in an acidic medium, impacting the intensity of the resulting fluorescence, as it directly influences the signal intensity of the reaction product (2,3-naphthotriazole). Adequate levels of DAN are required to ensure complete reaction with nitrite, thereby generating a measurable fluorescent signal. At substoichiometric concentrations of DAN, the reaction remains incomplete, resulting in diminished signal intensity relative to that of the nitrite present. Conversely, when DAN is supplied at or above the stoichiometric requirement, the reaction proceeds to completion and the signal reaches its maximum. Beyond this point, further increases in the DAN concentration do not enhance the response, as the signal becomes limited by the available nitrite.^29^

In order to assess the influence of this parameter in the proposed methodology, different DAN reagent concentrations ranging from 5 to 100 mg L^–1^ were studied, with the reaction time fixed at 10 min and the reaction temperature at 10 °C. As shown in Figure 3b, the analytical response tends to increase with concentration up to 50 mg L^–1^, after which the signal remains plateaued. To minimize background interference while maintaining sensitivity, particularly at trace-level nitrite detection, a concentration of 50 mg L^–1^ DAN was selected for subsequent studies, ensuring complete reaction and robust signal generation.

#### 3.3.3 Effect of Reaction Time

The duration of the reaction is a key factor in ensuring its completeness. In fluorometric systems, this parameter becomes even more relevant since fluorescent compounds may require a certain amount of time to stabilize and reach maximum emission intensity. If the reaction time is too short, then this stabilization may not occur. Furthermore, there is a decrease in the fluorescence intensity over time in conventional solution-based systems.^30^

According to Wiersma,^17^ the reaction between nitrite and the DAN reagent under acidic conditions is very rapid and typically begins to occur after 2 min. To determine the optimal reaction time, experiments were carried out at 2, 5, 10, 15, 20, 30, and 40 min. The parameters were maintained at a temperature of 10 °C and a DAN concentration of 50 mg L^–1^. The analytical response increased with increasing time up to 15 min (Figure 3c). Comparing the analytical responses obtained at 10 and 15 min using the paired *t*-test analysis, no statistically significant difference was found between these reaction times, as the calculated *t*-value (*t* = 0.82) was lower than the critical value (*t* = 4.30) at a 95% confidence level. Thus, at least a 10 min time is necessary to ensure the reaction goes to completion. For longer reaction times, a significant decrease in the analytical response was observed, which may be attributed mainly to the degradation of the reaction product leading to a decrease in the fluorescence intensity. Finally, aiming for a more efficient method, a reaction time of 10 min was selected for subsequent experiments, as it was the shortest time that provided reliable results, allowing good sample throughput.

The optimized conditions for the method were the following: 10 μL of nitrite standard solution or sample, 1 μL of 0.30 mol L^–1^ EDTA, and 2 μL of 50 mg L^–1^ DAN. The reaction was carried out for 10 min in a refrigerated environment at 10 °C. Subsequently, 2 μL of 0.50 mol L^–1^ sodium hydroxide was added before fluorescence measurement.

### 3.4 Interference Study

A broad range of ions present in natural water sample matrices can interfere with the reaction between nitrite and DAN.^16^ In this study, potential interfering ions commonly found in water samples were evaluated. The assessment was based on the recovery of 50 μg L^–1^ nitrite in samples individually fortified with potential interferents at different concentration levels. The experiments were performed using the following concomitants usually present in natural water matrices: K^+^, Ca^2+^, Mg^2+^, Fe^2+^, Fe^3+^, Zn^2+^, F^–^, NO_3_^–^, HCO_3_^–^, SO_4_^2–^, and SO_3_^2–^. Sodium and chloride ions were not evaluated as interferents, as they are inherently present in the reaction medium due to the use of HCl and NaOH.

In the proposed procedure, EDTA was added to the reaction medium between nitrite and DAN to prevent a potential interference. EDTA is a widely used complexing agent because of its high capacity to form stable complexes with a wide variety of metal ions. This helps avoid undesirable secondary reactions, stabilizes the reaction medium, and ensures greater precision and selectivity in the reaction.^31^

As observed from the results shown in Table 1, nitrite recoveries ranged from 86 to 114%. According to AOAC validation guidelines, recoveries within approximately 80–120% are considered acceptable for determinations at the trace level,^32^ indicating the absence of significant interference. Therefore, it can be concluded that this methodology exhibits a high tolerance to common matrix concomitants typically present in water samples.

**Table 1:** Nitrite Recovery Percentages in the Presence of Potential Interfering Ions at Different Concentration Levels

Interferent	RSD (%) *N* = 3	Studied Level (mg L^–1^)	NO_2_^–^ Recovery (%)
K^+^	2	1.0	102
	6	10.0	102
Ca^2+^	8	10.0	86
	4	90.0	104
Mg^2+^	8	5.0	109
	6	25.0	87
Fe^2+^	4	0.2	89
	3	1.0	95
Fe^3+^	8	0.2	107
	5	1.0	98
Zn^2+^	5	0.1	107
	7	1.0	93
F^–^	6	0.2	109
	7	1.5	114
NO_3_^–^	7	5.0	102
	2	50.0	95
HCO_3_^–^	3	20.0	98
	6	300.0	107
SO_4_^2–^	6	3.0	98
	3	250.0	98
SO_3_^2–^	2	0.5	110
	6	10.0	99

### 3.5 Analytical Figures of Merit

The analytical performance parameters for the proposed procedure under optimal conditions were evaluated, including the linear range for the analytical calibration, limit of detection (LOD), limit of quantification (LOQ), and precision (repeatability, as relative standard deviation %). The calibration curve, based on nitrite detection, was linear over the concentration range of 10–150 μg L^–1^ with correlation coefficients (r) superior to 0.996 (Figure 4).

**Figure 4:**
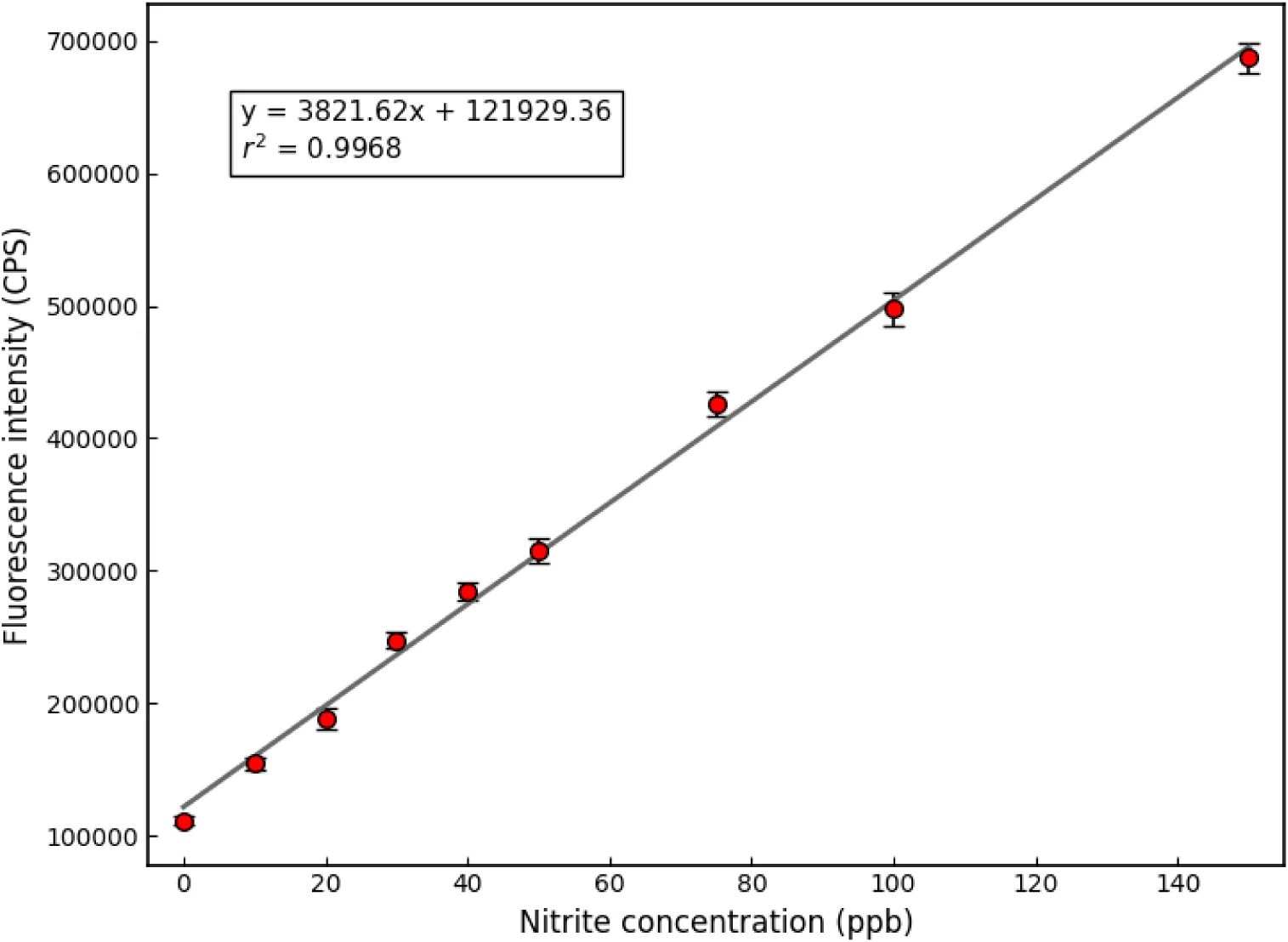
Calibration curve for nitrite determination based on fluorescence intensity using the proposed method (*N* = 3).

Precision, evaluated through repeatability at a nitrite concentration of 50 μg L^–1^ and expressed as relative standard deviation (RSD%, *N* = 7), was 3.5%, indicating good precision. The LOD was 0.35 μg L^–1^ and the LOQ was 1.17 μg L^–1^.

Furthermore, Table 2 shows a comparison of the proposed approach with others reported in the literature based on nitrite determination in aqueous samples using paper-based analytical platforms, including approaches based on the conventional Griess assay. A comparison of these results shows that the LOD and LOQ values obtained in the proposed procedure are lower than those reported in the literature, indicating the good sensitivity of the method.

**Table 2:** Analytical Performance of the Proposed Method in Comparison with Literature Reports

Analytical method	Sample	Sample volume (μL)	Reagents volume (μL)	LOD (μg L^–1^)	LOQ (μg L^–1^)	Reference
Microfluidic Griess assay/Colorimetry	Mineral water	15.0	10.0	300.0	1100.0	^33^
Microfluidic Griess assay/Colorimetry	Water	40.0	12.0	100.0	700.0	^34^
Microfluidic DHBPTz assay/Colorimetry^a^	Water	10.0	2.0	59.8	101.2	^35^
*N*-(1-naphthyl)ethylenediamine-functionalized cellulose bridge/Colorimetry	Sea water	15.0	5.0	10.1	40.9	^36^
DAN–AuNCs-based paper/Colorimetry	Water	5.0	20.0	2.3	8.7	^37^
Microfluidic Griess assay/Colorimetry	Sea water	15.0	5.0	2.3	7.8	^38^
Microfluidic Griess assay/Colorimetry	Sea water	30.0	1.2	0.61	-	^39^
GNRs–GNPs-based paper Griess assay/Fluorometry^b^	Water and meat	10.0	100.0	0.46	-	^40^
Hydrophobic substrate-based DAN assay/Fluorometry	Water	10.0	5.0	0.35	1.17	This work

a DHBPTz: 1,2-dihydro-3,6-bis­(3,5-dimethyl-1H-pyrazol-1-yl)-1,2,4,5-tetrazine.

b GNRs-GNPs: gold–gold nanoparticles; AuNCs: gold nanoclusters.

One of the main advantages of using fluorometry is its greater sensitivity compared with colorimetric assays. This improved sensitivity can be attributed to the use of fluorometric detection, which is generally more sensitive than colorimetric assays, such as those based on the Griess reaction. However, it is important to note that differences in the experimental conditions can influence direct comparisons.

Regarding paper-based devices based on hydrophilic cellulose substrates, although they present several advantages, a potential limitation is the possible loss of sensitivity due to reactions occurring on the cellulose matrix substrate (whether colorimetric or luminescent) since the reagents and reaction products are embedded within the white cellulose matrix. A key strength of this study is the incorporation of a hydrophobic cellulose substrate, which confines the fluorescent reaction to the material’s surface rather than its bulk. This surface-restricted reaction markedly amplifies the fluorescence emission, thereby substantially enhancing the overall analytical sensitivity of the assay. This contributes to minimizing the reduction in fluorescent signal observed on conventional hydrophilic substrates and, together with the high sensitivity of the DAN fluorimetric reaction, explains the analytical performance achieved by the proposed device.

In addition, the proposed methodology retains the inherent advantages of a paper-based analytical platform, such as analytical miniaturization, reduced reagent consumption, lower waste generation, and shorter analysis time. To enable direct fluorimetric measurements using conventional instrumentation without the need for sample handling steps or transfer to analysis vessels, such as the microcuvettes commonly used.

### 3.6 Accuracy and Analysis of Water Samples

Accuracy was assessed using a recovery study for nitrite in different water samples, which were fortified at two concentration levels: 10 μg L^–1^ and 50 μg L^–1^. The results are shown in Table 3. The recoveries values ranged from 83% to 115% and remain within the AOAC-acceptable criteria for the tested concentration level (μg L^–1^),^32^ which confirms the accuracy of the proposed procedure.

**Table 3:** Measured Values and Recoveries Obtained Using the Proposed Methodology for the Analysis of Water Samples Collected from Different Sampling Sites (*N* = 3)

Sample	Sampling site	Added concentration (μg L^–1^)	Measured concentration (μg L^–1^) ± s (*N* = 3)	Recovery (%) ± s (*N* = 3)
Mineral water	Commercial bottled water	0	<LOQ	-
		10	10.0 ± 0.3	100 ± 3
		50	53.4 ± 2.4	107 ± 5
Tap water	University of Vigo	0	<LOQ	-
		10	9.2 ± 0.2	92 ± 2
		50	52.3 ± 1.6	105 ± 3
Public drinking fountain	Vigo, Spain	0	<LOQ	-
		10	10.1 ± 0.5	101 ± 5
		50	47.1 ± 2.5	94 ± 5
Wall-mounted public drinking fountain	Vigo, Spain	0	<LOQ	-
		10	9.4 ± 0.6	94 ± 6
		50	41.5 ± 0.1	83 ± 1
Natural spring water fountain	Vigo, Spain	0	6.1 ± 0.4	-
		10	16.8 ± 0.5	107 ± 5
		50	49.1 ± 0.5	86 ± 1
River water	Vigo, Spain	0	14.0 ± 1.2	-
		10	22.6 ± 0.8	86 ± 8
		50	57.9 ± 0.9	86 ± 2
Reservoir water	Vigo, Spain	0	29.3 ± 0.7	-
		10	39.0 ± 0.1	97 ± 1
		50	87.0 ± 0.9	115 ± 2

To demonstrate the applicability of the proposed approach, different water samples were analyzed, including mineral water, tap water, public drinking fountain, natural spring water fountain, wall-mounted public drinking fountain, river water, and reservoir water. Only three of the analyzed samples showed quantifiable concentrations, ranging from 6.1 to 29.3 μg L^–1^. According to the European Union Directive on the quality of water intended for human consumption,^5^ the maximum allowed limit for nitrite is 500.0 μg L^–1^, a value much higher than those found in both drinking water and nondrinking water samples.

### 3.7 Green Analytical Metrics

The methodology developed was evaluated based on the ten principles of green sample preparation (GSP) using the AGREEprep open-access software.^41^ This evaluation considers characteristics of analytical procedures, such as the use of safer and less toxic solvents and reagents, the application of sustainable materials, the minimization of waste and energy consumption, and the promotion of automation, among others. The estimate provides a score ranging from 0 to 1, where results closer to 1 indicate a higher conformity with the principles established by GSP.^41,42^

This procedure, based on the confinement into a microdroplet of the fluorimetric reaction of nitrite with DAN in an acidic medium, supported onto a hydrophobic cellulose substrate, achieved a score of 0.77 (Figure 5). This assessment is based on a systematic analysis of the ten GSP principles. The results were highly favorable at the principles 2, 4, and 5, which refer to the use of small volumes of solvents and reagents, only 5 μL per sample, waste minimization with very low residue generation, and the reduction of sample, chemical, and material consumption, working with just 10 μL of sample. Although DAN is acknowledged for its high toxicity, its use in very small volumes (2 μL) contributes significantly to reducing occupational exposure risks and environmental impact. These outcomes highlight the low cost, safety, and reduced environmental impact of the proposed method.

**Figure 5:**
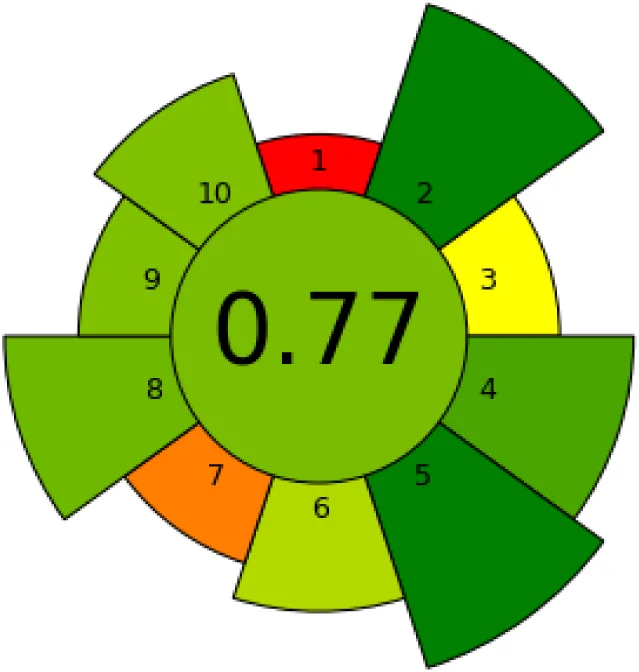
AGREEprep assessment result of the analytical procedure based on the reaction of nitrite with 2,3-diaminonaphthalene on a hydrophobic cellulose substrate.

Criteria 6, 8, 9, and 10 scored above average, relating to the overall speed of the sample preparation procedure, the highest possible energy efficiency, the use of greener and simpler instrumental techniques, and the basic risks associated with the procedure. These criteria received quite satisfactory evaluations with individual scores above 0.50.

The less favorable criteria were criterion 1, which recommends in situ sample preparation; however, in this study, sample preparation was conducted in the laboratory. Criterion 3 assesses the use of sustainable, reusable, and renewable materials; since the hydrophobic cellulose substrate is a single-use device, it scored lower, although the waste generated is minimal because of its small size. Finally, criterion 7, which aims to promote automation, also received a lower score because all steps were performed manually.

These limitations highlight avenues for future development. Potential integration with portable detection systems, investigation of reusable hydrophobic substrates, and the implementation of automated procedures can further enhance the platform’s sustainability, broadening its applicability in environmental monitoring strategies and in situ analyses.

## 4 Conclusions

The proposed procedure for determining nitrite based on the fluorometric reaction with 2,3-diaminonaphthalene using hydrophobic cellulose substrates, both as a reaction medium and detection platform, proved to be efficient and sensitive, as evidenced by the evaluation of analytical parameters with low LOD, LOQ, and RSD%, and in comparison with widely adopted reference methods that exhibited higher limits of quantification than this methodology. Furthermore, an innovative method was developed for which no previous studies were found in the literature combining hydrophobic cellulose substrates with fluorometric reaction using the DAN reagent.

The microscale procedure on the hydrophobic surface facilitated the detection of strong fluorometric signals even when using small volumes, demonstrating that the approach goes beyond the simple reduction of reagent volumes. In this context, the high value of the AGREEprep green analysis metric (0.77) highlights that the innovative method enables an analytical approach aligned with the principles of green analytical chemistry, reducing the level of reagent consumption and waste generation.

In addition, replacing conventional fluorometric cuvettes with a cellulose-based substrate as the detection platform, without any loss of sensitivity, provides a practical, low-cost, and accessible alternative while maintaining adequate analytical performance for routine analysis and environmental monitoring.

## Supplementary Material



## Notes

Photographs of the experimental setup and microdroplet stability (Figure S1) and emission spectra of the reaction product (Figure S2) (PDF)
