# Supplementary material for: A Microdroplet Fluorescence Method for the Determination of Nitrite in Water Using a Hydrophobic Cellulose Substrate Platform
Source: ACS Omega. 2026 Sep 29;11(40):61358–66. doi: 10.1021/acsomega.6c06452 (PMC13670833; doi:10.1021/acsomega.6c06452)
Supplement: Supplementary file 1 [file ao6c06452_si_001.pdf]

A microdroplet fluorescence method for the determination of nitrite in water using a hydrophobic cellulose substrate platform

Júlia C. Romero[b] §, Vanesa Romero[b*], Carlos Bendicho[b], Fábio A. C. Amorim[a*]

a - Departamento de Ciências Exatas, Universidade Estadual de Santa Cruz, Campus Soane Nazaré de Andrade, Rodovia Jorge Amado, 45662-900 Ilhéus, Brazil.
b - Centro de Investigación Mariña, Departamento de Química Analítica y Alimentaria, Grupo QA2, Edificio CC Experimentais, Universidade de Vigo, Campus de Vigo, As Lagoas Marcosende, 36310 Vigo, Spain;

Corresponding author: facamorim@uesc.br and vromero@uvigo.gal

Supporting Information

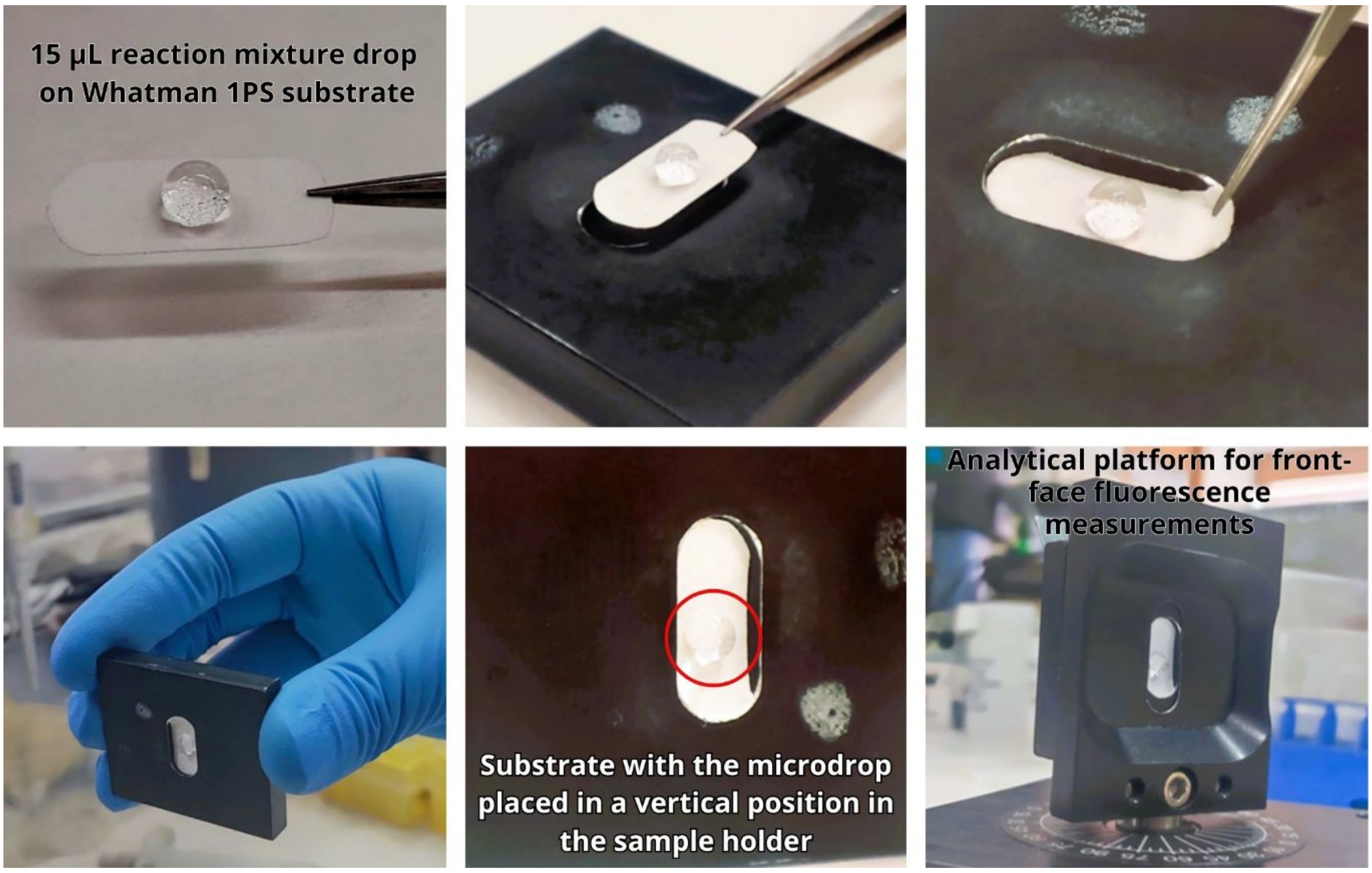

Figure S1. Photograph of the front-surface fluorescence measurement setup showing the stable microdroplet suspended on the vertical hydrophobic cellulose substrate.

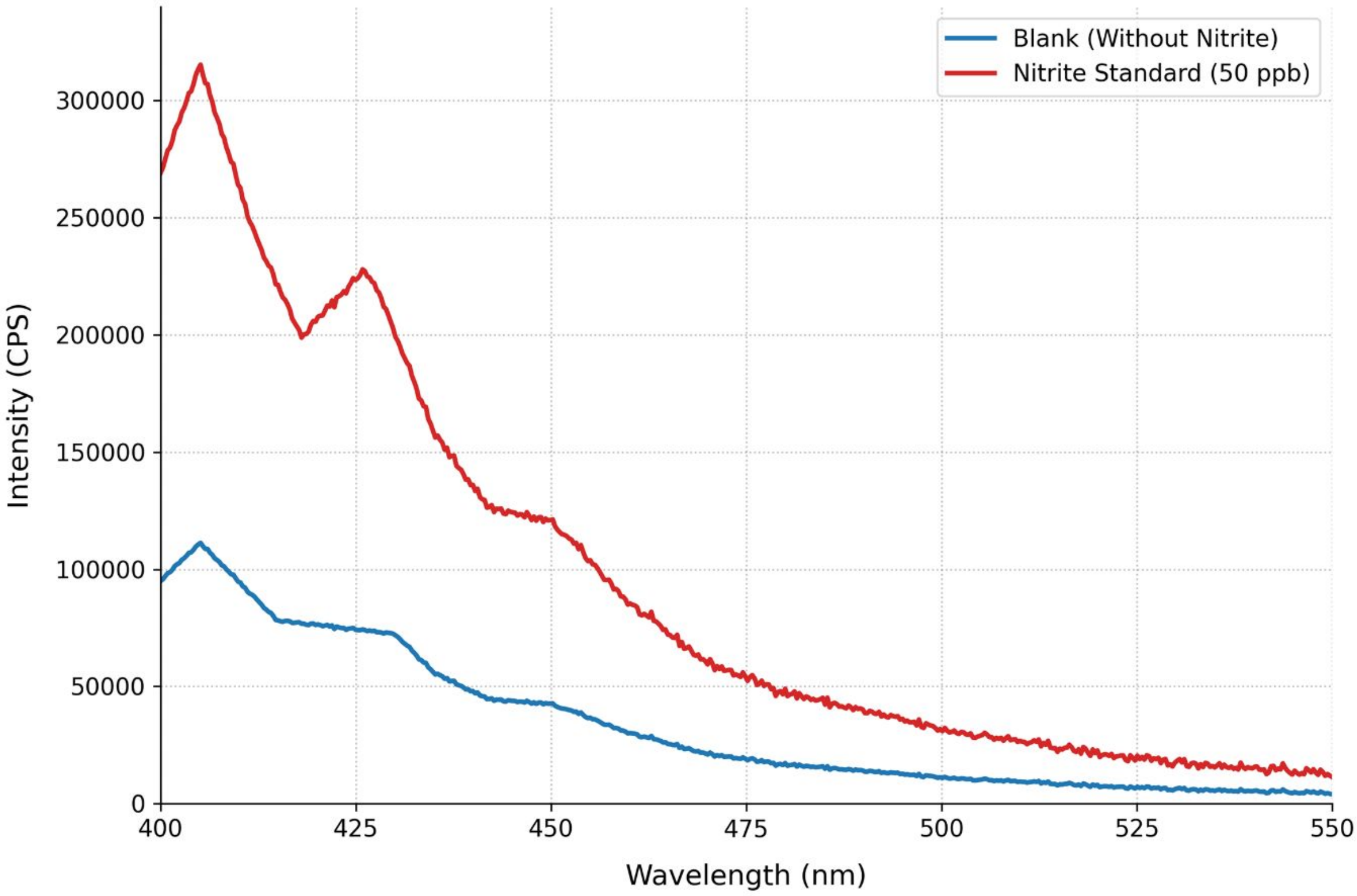

Figure S2. Fluorescence emission spectra for the analytical blank and after the addition of 50 μg L^{-1} of nitrite.
